# Real-world HbA_1c_ changes and prescription characteristics among type 2 diabetes mellitus patients initiating treatment with once weekly semaglutide for diabetes

**DOI:** 10.1007/s40200-023-01341-y

**Published:** 2023-11-27

**Authors:** Monica Frazer, Caroline Swift, Andrew Sargent, Michael Leszko, Erin Buysman, Noelle N. Gronroos, Sara Alvarez, Tyler J. Dunn, Josh Noone, Cory L. Gamble

**Affiliations:** 1 QualityMetric, Johnston, RI USA; 2 Novo Nordisk Inc., Plainsboro, NJ USA; 3 Optum, 11000 Optum Circle Eden Prairie, Eden Prairie, MN 55344 USA

**Keywords:** GLP-1 RA, Type 2 Diabetes, HbA_1c_, Semaglutide

## Abstract

**Purpose:**

The purpose of this study was to evaluate patient, prescriber, and dose characteristics and evaluate changes in glycated hemoglobin (HbA_1c_) for patients prescribed once weekly semaglutide for diabetes (OW sema T2D).

**Methods:**

This study was a retrospective claims-based study using the Optum Research Database. The sample included adult patients who had at least one claim for OW sema T2D between Jan 1, 2018, and Dec 31, 2019, were continuously enrolled in the health plan and had a diagnosis of type 2 diabetes (T2DM) during the pre-index or post-index periods. Demographic and clinical characteristics of patients using OW sema T2D were collected, as were the dose and prescriber specialty and the change between pre-index and post-index HbA_1c_ measures was calculated. Results were stratified by the latest pre-index HbA_1c_ measurement (HbA_1c_ greater than or equal to 9.0%, uncontrolled vs. HbA_1c_ less than 9%, controlled). Statistical comparisons between HbA_1c_ groups were conducted.

**Results:**

Most patients, 76.3%, were prescribed a 0.25/0.50 mg dose of OW sema T2D. Patients had an overall decrease in HbA_1c_ of 0.8% and patients with uncontrolled diabetes had a greater reduction in mean HbA_1c_ compared to those with controlled diabetes (-2.1% vs. -0.3%, p < 0.001). Most patients had their index dose of OW sema T2D prescribed by endocrinologists (27.6%) primary care providers (24.6%) and internal medicine providers (21.6%).

**Conclusions:**

OW sema T2D is an effective real-world T2DM treatment. Future research should further investigate real-world use patterns of this medication.

## Introduction

Type 2 Diabetes (T2DM) is a critical public health challenge worldwide [1]. T2DM is a consequence of insulin resistance – where cells stop responding appropriately to insulin – and defective insulin secretion by pancreatic β cells [2]. These factors disrupt glucose homeostasis, and symptoms include frequent urination, thirst, blurred vision, numbness in the hands and feet, and fatigue among others [3]. Because symptoms develop slowly over years, patients often don’t notice them [3]. Risk factors include being overweight or obese, having an age over 45, having a family history of diabetes, and physical inactivity [3]. Diabetes is associated with microvascular, macrovascular, and neurological complications [4–6]. Since 1980, the prevalence of T2DM in adults has increased or stayed the same in every region of the world, and the number of adults living with diabetes has quadrupled [7]. In the United States, an estimated 30.2 million Americans have diabetes, 90–95% of whom have T2DM [8].

For patients with T2DM, proper treatment and intervention is important. Guidelines recommend that people with diabetes receive medical care from a collaborative, integrated team [4–6, 9, 10]. Glycated hemoglobin (HbA_1c_) is a measure of glycemic control over the previous 90 days and has been shown to predict diabetes related complications; regular testing is recommended for patients with diabetes [11]. Glycemic control has been shown to slow the progression of complications such as diabetic retinopathy, nephropathy, and neuropathy in patients with T2DM [12]. According to Stratton et al., a reduction in HbA_1c_ of 1% is associated with a reduction in risk of 21% for any diabetes related end point [13]. Current guidelines for HbA_1c_ from the American Diabetes Association encourage patients reach and maintain optimal HbA_1c_ levels [11]. Lowering HbA_1c_ to below 7% has been shown to reduce complications of diabetes and is a goal for many adult patients [4–6, 10, 11].

Glucagon-like peptide-1 receptor agonists (GLP-1 RAs) are a class of antihyperglycemic drugs that were first approved for treatment of patients with T2DM in 2005 [14]. GLP-1 RAs have been shown to decrease HbA_1c_ levels by at least 1% and to have positive impacts on weight, blood pressure, and lipid levels [14]. GLP1- RAs are recommended for patients who need to minimize the risk of hypoglycemia and to help promote heathy weight management [15, 16]. Once-weekly semaglutide for diabetes (OW sema T2D) is a GLP-1 RA administered weekly as a subcutaneous injection first approved by the FDA in 2017. In the Semaglutide Unabated Sustainability in Treatment of Type 2 Diabetes (SUSTAIN) clinical trials, OW sema T2D was effective at lowering HbA_1c_ levels in patients with T2DM [17–27]. Across the SUSTAIN trials, a 1.0 mg dose of OW sema T2D was shown to reduce HbA_1c_ by 1.5–1.8% after 30–56 weeks [28]. Additionally, patients using OW sema T2D reduced their body weight significantly more than patients who used the DPP-4, SGLT-2, daily subcutaneous GLP-1 RA, once weekly subcutaneous GLP-1 RA and insulin medications tested in the trials [28]. Recently, results from the SUSTAIN-FORTE trial showed that patients on a 2.0 mg dose of OW sema T2D had their HbA_1c_ levels reduced by an average of 2.1%.^27^ Dosing instructions for OW sema T2D recommend an initial dose of 0.25 mg weekly and that this dose be increased to 0.50 mg weekly after 4 weeks [29]. After 4 weeks at the 0.50 mg dose, a 1.0 mg weekly dose may be used if the patient needs further glycemic control [29]. At the time this study was conducted, the 1.0 mg weekly dose was the maximum dose; a 2.0 mg weekly dose was approved in 2022 and this dose can be prescribed if additional glycemic control is needed [27].

OW sema T2D has a safety profile that is similar to other GLP-1 RAs [14]. OW sema T2D has been shown to induce mild and transient gastrointestinal (GI) disturbances and to increase the risk of cholelithiasis [14]. Due to its robust glucose lowering effect, patients with potentially unstable diabetic retinopathy using GLP-1 RAs should be closely monitored [14]. Providers can help patients manage adverse events (AEs), with mild GI AEs (e.g., nausea) managed with recommended lifestyle changes, dose-dependent AEs managed by modifying the dosing schedule, and more severe AEs (e.g., severe diarrhea, persistent vomiting) evaluated and treated medically [30]. Additionally, older patients should be evaluated for sarcopenia and resistance training is recommended as necessary to reduce the risk of worsening this condition with weight loss [30].

Real-world evidence (RWE) can augment the knowledge base of clinical trials and helps build understanding around the effectiveness of diabetes interventions in a real-world setting [31]. Williams et al. conducted a real-world observational study of patients initiating treatment with OW sema T2D in Wales [32] and found that OW sema T2D reduced HbA_1c_ significantly. Visaria et al. also examined HbA_1c_ changes in 1,888 patients initiating therapy with OW sema T2D between December 1, 2017 and April 30, 2019 in a cohort of commercially insured and Medicare Advantage patients [33] and found a mean HbA_1c_ change in the overall population of 0.9%.

This study builds on knowledge from clinical trials and RWE studies in assessing the effectiveness of OW sema T2D in a large sample of patients initiating OW sema T2D over a 2-year period. The objectives of this study were to (1) evaluate patient characteristics prior to initiation of OW sema T2D along with prescriber characteristics and index and maintenance dosages and (2) evaluate changes in HbA_1c_ among patients prescribed OW sema T2D, including among those who were prescribed OW sema T2D for at least 90 days (persistent patients).

## Methods

### Design

This was a retrospective study using medical claims, pharmacy claims, and enrollment information from Jan 1, 2017, to Dec 31, 2020 (study period). The data source for this study was the Optum Research Database (ORD), one of the largest and most complete real world data sources for US patients. Data in ORD are from commercial and Medicare Advantage health plan members, are fully deidentified, and are Health Insurance Portability and Accountability Act (HIPAA) compliant. Data include medical and pharmacy claims data (including linked enrollment) from 1993 to present on more than 73 million lives. Medical claims for this investigation were identified by using International Classification of Diseases, 10th Revisions, Clinical Modification diagnosis codes and procedure codes, Current Procedural Terminology codes, Healthcare Common Procedure Coding System codes, place of service codes, provider specialty codes, and revenue codes. Relevant outpatient pharmacy claims were identified using National Drug Code. This study did not require Institutional Review Board approval or waiver of authorization as no identifiable protected health information was accessed.

### Study sample

Patients were included in the study sample if they had at least one claim for OW sema T2D (date of first claim = index) between Jan 1, 2018 and Dec 31, 2019 (identification period), were at least 18 years old in the year of the index date, were continuously enrolled in the health plan for at least 12 months prior to and including the index date (pre-index period) and 12 months following the index date (post-index period), and had at least 1 claim indicating a diagnosis of T2DM during the pre-inde or post-index periods. Patients with at least one claim indicating pregnancy during the pre-index or post-index periods were excluded as were patients with missing age, gender or region information and patients for which there was no HbA_1c_ value recorded during the pre-index period.

### Study variables

Data on patient demographic and clinical characteristics were collected from claims including age, gender, insurance type and geographic region. The Charlson comorbidity score was calculated based on the presence of diagnosis codes on medical claims in the pre-index period [34, 35]. General comorbid conditions were defined using the Clinical Classifications Software managed by the Agency for Healthcare Research and Quality (AHRQ) [36]. Data on pre-index medication classes were collected by analyzing the proportion of patients with at least one pharmacy claim for American Hospital Formulary Service (AHFS) medication classes. Data on pre-index diabetes medication classes was obtained by examining the proportion of patients with at least one pharmacy claim for an antidiabetic agent (biguanides, sodium-glucose cotransporter-2 inhibitors [SGLT-2s], GLP-1 RAs, dipeptidyl peptidase 4 inhibitors [DPP-4s], thiazolidinediones [TZDs], sulfonylureas, and insulin) during the pre-index period. Additional agents including alpha-glucosidase inhibitors, meglitinides, bile acid sequestrants, dopamine-2 agonists, and amylin mimetics were assessed but not included in this analysis.

Prescription data for patients prescribed OW sema T2D was collected. The dose on the first pharmacy claim (index claim) for OW sema T2D during the identification period was the index dose, and the maintenance dose was the dose with the largest proportion of days covered starting on the index date through the end of follow-up. Data on prescriber specialty was obtained using the specialty recorded on the index pharmacy claim. Data collected related to HbA_1c_ measures included the change from the last HbA_1c_ value measured during the pre-index period and the last HbA_1c_ value measured during the post-index period. To identify HbA_1c_ results in patients persistent with OW sema T2D, HbA_1c_ results recorded at least 90 days after the index date in patients with at least 90 days of continuous therapy were assessed. Discontinuation was defined as having a gap of at least 60 days in OW sema T2D treatment and the date of discontinuation was defined by the date by which the last prescription for OW sema T2D would run out prior to the gap in therapy.

### Analysis

Results were stratified by the latest pre-index HbA_1c_ measurement. The Healthcare Effectiveness Data and Information Set (HEDIS) defines poor glycemic control as an HbA_1c_ of greater than or equal to 9% [37]. For brevity, patients with an HbA_1c_ greater than or equal to 9.0% were termed “uncontrolled” and those with an HbA_1c_ of less than 9% were termed “controlled”. The focus of the analysis was on the uncontrolled cohort and the “controlled” cohort was included for completeness. HbA_1c_ change results were presented in the subset of patients with HbA_1c_ values in the pre-index and post-index periods overall and among those who were persistent. Numbers and percentages were provided for categorical variables, means and standard deviations were provided for continuous variables. Statistical comparisons between groups were conducted with t-tests for continuous variables, and with chi-square tests for dichotomous variables. For comparisons of change from pre-index to post-index HbA_1c_ values within patients, students t-tests were used.

## Results

### Patient sample

**Fig. 1:**
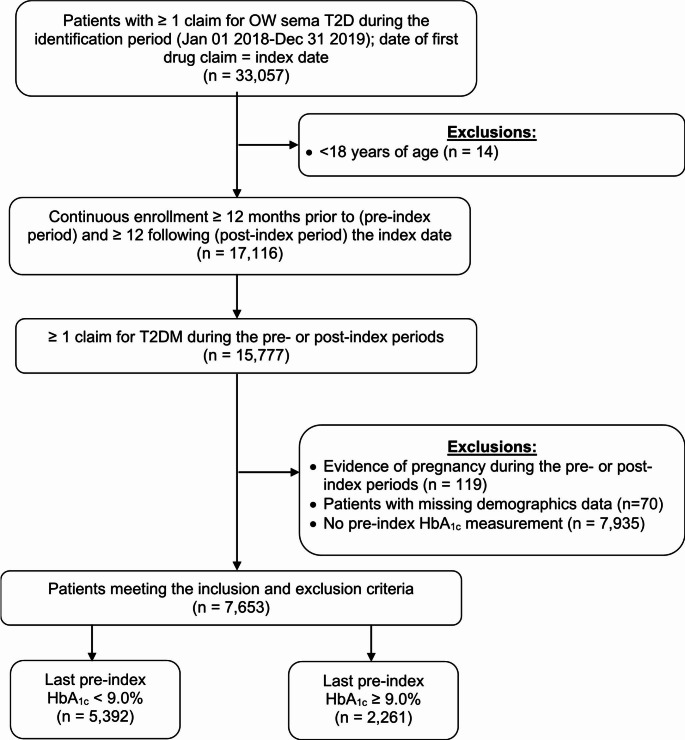
Patient sample selection

The study sample was comprised of 7,653 patients (Fig. 1). 2,261 patients (29.5%) had a last pre-index HbA_1c_ measurement of greater than or equal to 9% (uncontrolled) and 5,392 patients (70.5%) had a last pre-index HbA_1c_ of less than 9% (controlled).

### Pre-index patient characteristics

Pre-index patient demographic and clinical characteristics are described in Table 1. Overall, the mean age of patients in this sample was 58.7 (SD 11.4) years; most patient data came from the Southern US (66.9%), followed by the Midwest (12.0%) and most patients (62.4%) had commercial insurance (Table 1). The mean age of patients in the controlled group was significantly higher than the uncontrolled group (59.4 years vs. 57.2 years, p < 0.001). There were fewer males in the controlled group compared to the uncontrolled group (46.8% vs. 52.7%, p < 0.001), and patients in the controlled group were more likely than those in the uncontrolled group to be covered by Medicare (38.4% vs. 35.4%, p = 0.013).

**Table 1:** Patient demographics and clinical characteristics

	Total (n = 7,653)	Pre-index HbA_1c_ ≥ 9% (n = 2,261)	Pre-index HbA_1c_ < 9% (n = 5,392)	p-value
**Age, mean (SD)**	58.7 (11.4)	57.2 (11.4)	59.4 (11.3)	< 0.001
**Age group, n (%)**				
18–39 years	396 (5.2)	146 (6.5)	250 (4.6)	0.001
40–64 years	4,761 (62.2)	1,485 (65.7)	3,276 (60.8)	< 0.001
65–74 years	1,942 (25.4)	512 (22.6)	1,430 (26.5)	< 0.001
75 + years	554 (7.2)	118 (5.2)	436 (8.1)	< 0.001
**Male gender, n (%)**	3,716 (48.6)	1,192 (52.7)	2,524 (46.8)	< 0.001
**Insurance type, n (%)**				
Commercial	4,779 (62.4)	1,460 (64.6)	3,319 (61.6)	0.013
Medicare	2,874 (37.6)	801 (35.4)	2,073 (38.4)	0.013
**Region, n (%)** ^**1**^				
Northeast	785 (10.3)	217 (9.6)	568 (10.5)	0.218
Midwest	917 (12.0)	279 (12.3)	638 (11.8)	0.533
South	5,119 (66.9)	1,502 (66.4)	3,617 (67.1)	0.581
West	831 (10.9)	263 (11.6)	568 (10.5)	0.159
**Quan-Charlson comorbidity index score, mean (SD)**	1.5 (1.7)	1.5 (1.7)	1.5 (1.7)	0.298
**Top AHRQ comorbid conditions, n (%)**				
Lipid metabolism disorder	6,778 (88.6)	1,971 (87.2)	4,807 (89.2)	0.013
Diabetes mellitus without complications	6,678 (87.3)	1,909 (84.4)	4,769 (88.5)	< 0.001
Hypertension	6,555 (85.7)	1,933 (85.5)	4,622 (85.7)	0.797
Diabetes mellitus with complications	6,158 (80.5)	1,981 (87.6)	4,177 (77.5)	< 0.001
Other nutritional, endocrine, or metabolic disorder	5,630 (73.6)	1,568 (69.4)	4,062 (75.3)	< 0.001
**Valid n** ^2^	6,042	1,719	4,323	
Pre-index HbA_1c_^3^, mean (SD)	8.2 (1.8)	10.5 (1.3)	7.3 (0.9)	< 0.001

^1^ Patients with region of other are not presented due to small sample size

^2^Patients with an HbA_1c_ measurement in both the pre-index and post-index periods

^3^Based on most recent HbA_1c_ value measured during the pre-index period or on the index date

SD, standard deviation; AHRQ, Agency for Healthcare Research and Quality

### Comorbid conditions

The overall mean Charlson comorbidity index score was 1.5 (SD 1.7). The mean Charlson score was similar for patients in the controlled and uncontrolled groups. The most common AHRQ comorbidities for patients were disorders of lipid metabolism (88.6%), hypertension (85.7%), and diabetes mellitus with complications (80.5%). Patients with uncontrolled diabetes were more likely to have diabetes mellitus with complications than those with controlled diabetes (87.6% vs. 77.5%, p < 0.001).

### Pre-index medications

**Fig. 2:**
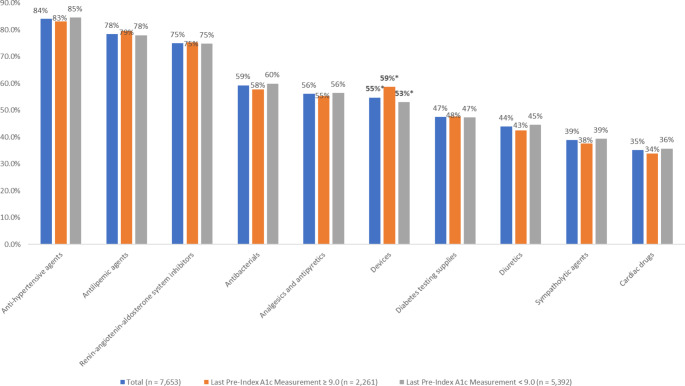
Top 10 AHFS Medication Classes Prescribed in the pre-index period (including drugs filled on the index date)

**Fig. 3:**
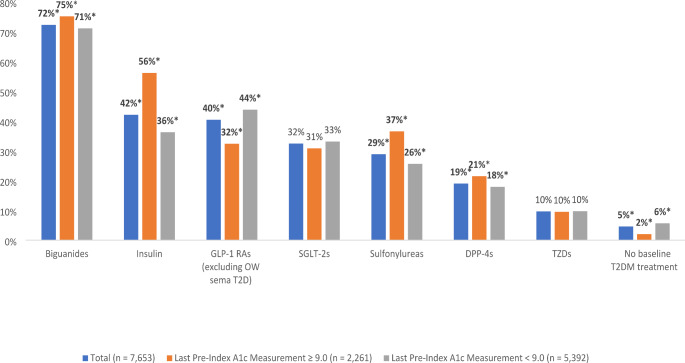
Diabetes drugs filled in the pre-index period excluding OW sema T2D (including drugs filled on the index date)

During the pre-index period, patients were prescribed an average of 13.6 (SD 6.4) different classes of medications and 15.5 (SD 7.5) different compounds. The most commonly prescribed AHFS classes included anti-hypertensive agents (84.2%), antilipemic agents (78.4%), and renin-angiotensin-aldosterone system inhibitors (75.0%) (Fig. 2). The most commonly prescribed diabetes medications include: biguanides (72.3%), insulin (42.1%), and GLP-1 RAs excluding OW sema T2D (40.4%) (Fig. 3). Patients with uncontrolled diabetes were less likely than those with controlled diabetes to have pre-index experience with GLP-RAs (excluding OW sema T2D) (32.4% vs. 43.8%, p < 0.001). Patients with uncontrolled diabetes were also less likely than those with controlled diabetes to have no pre-index treatment (2.0% vs. 5.7%, p < 0.001). During the pre-index period, patients with uncontrolled diabetes were more likely than those with controlled diabetes to have been prescribed biguanides (75.1% vs. 71.1%, p < 0.001), DPP-4s (21.5% vs. 17.9%, p < 0.001), sulfonylureas (36.5% vs. 25.6%, p < 0.001), and insulin (56.2% vs. 36.2%, p < 0.001).

### Dose and prescriber characteristics

Index dose and prescriber characteristics are described in Table 2. It is not possible to distinguish between patients prescribed a 0.25 mg dose and those prescribed a 0.50 mg dose as they are dispensed in the same pre-filled injectable pen and the appropriate dose is selected at the time of injection. For initial prescriptions, 76.3% of all patients were initially prescribed a 0.25/0.50 mg dose of OW sema T2D while 23.7% were prescribed an initial 1.0 mg dose. The percentage of patients prescribed a 0.25/0.50 mg maintenance dose went down to 61.2% during the follow-up period while the percentage of patients prescribed a prescribed a 1.0 mg maintenance dose grew to 38.8%. Stratifying by baseline HbA_1c_, a significantly higher proportion of patients with uncontrolled diabetes were prescribed an initial 0.25/0.5 mg dose than those with controlled diabetes (80.4% vs. 74.6%, p < 0.001). while 63.5% of patients in the uncontrolled group and 60.2% of patients in the controlled group were prescribed a maintenance dose of 0.25/0.50 (p = 0.007). Overall, 27.6% of patients had their index dose of OW sema T2D prescribed by an endocrinologist while 24.6% had their index dose prescribed by a primary care physician and 21.6% of patients had their index dose prescribed by an internal medicine provider. The remaining patients were prescribed their index dose by cardiologists (0.5%), other providers (19.5%) and providers of unknown specialty (6.3%). A higher percentage of patients with uncontrolled diabetes received their index OW sema T2D prescription from primary care providers than those with controlled diabetes (27.5% vs. 23.4%, p < 0.001). Compared to those with controlled diabetes, a lower percentage of patients with uncontrolled diabetes received their index OW sema T2D prescription from internal medicine providers (19.4% vs. 22.4%, p = 0.003) and endocrinologists (25.5% vs. 28.5%, p = 0.008).

**Table 2:** Dose and provider specialty

Index OW sema T2D variable	Total (n = 7,653)	Last Pre-index HbA_1c_ Measurement ≥ 9.0 (n = 2,261)	Last Pre-index HbA_1c_ Measurement < 9.0 (n = 5,392)	p-value
**Initial daily dose of OW sema T2D, n (%)**				
0.25/0.5 mg	5,841 (76.3)	1,818 (80.4)	4,023 (74.6)	< 0.001
1.0 mg	1,812 (23.7)	443 (19.6)	1,369 (25.4)	< 0.001
**Prescriber specialty on pharmacy claim**^1^, **n (%)**				
Primary care	1,882 (24.6)	622 (27.5)	1,260 (23.4)	< 0.001
Internal Medicine	1,649 (21.6)	439 (19.4)	1,210 (22.4)	0.003
Endocrinologist	2,112 (27.6)	577 (25.5)	1,535 (28.5)	0.008
Other	1,492 (19.5)	460 (20.3)	1,032 (19.1)	0.225
Unknown	479 (6.3)	158 (7.0)	321 (6.0)	0.088
**Maintenance dose of OW sema T2D, n (%)**				
0.25/0.5 mg	4,683 (61.2)	1,436 (63.5)	3,247 (60.2)	0.007
1.0 mg	2,970 (38.8)	825 (36.5)	2,145 (39.8)	0.007

^1^Patients with a prescriber of neurologist or cardiologist are not presented due to small sample size, the primary care category consists of Clinic/Family Practice, Family/General Practice, OB/GYN, Pediatrics, Geriatrics; the other category includes specialties that are not part of any other category

### HbA_1c_ changes

HbA_1c_ changes among those who had both pre-index and post-index HbA_1c_ values (n = 6,042) are summarized in Table 3. The mean change between the latest pre-index measure and the latest post-index measure of HbA_1c_ was − 0.8% (SD 1.6) with 38.5% of patients having a decrease in HbA_1c_ of greater than or equal to 1%. Stratifying by baseline HbA_1c_, patients with uncontrolled diabetes had a statistically significant mean HbA_1c_ change of -2.1% (SD 2.0) (p < 0.001). Almost three-quarters (73.2%) of patients with uncontrolled diabetes had a decrease in HbA_1c_ of greater than or equal to 1% compared to 24.7% of patients with controlled diabetes (p < 0.001). Among patients who were persistent with OW sema T2D and had a valid HbA_1c_ measurement during the persistent treatment period, (n = 3,748), the mean decrease in HbA_1c_ was − 1.0% (SD 1.5) and 42.3% had a decrease in HbA_1c_ of greater than or equal to 1%. Persistent patients with uncontrolled diabetes had a statistically significant mean HbA_1c_ change of -2.5% (SD 1.8), (p < 0.001). Most (81.8%) persistent patients with uncontrolled diabetes had a decrease in HbA_1c_ of greater than or equal to 1%.

**Table 3:** Changes in HbA_1c_

		Total (n = 7,653)	Pre-index HbA_1c_ ≥ 9% (n = 2,261)	Pre-index HbA_1c_ < 9% (n = 5,392)	p-value
**Patients with ≥ 1 HbA**_**1c**_ **measurement in both pre- and post-index periods, n (%)**		6,042 (79.0)	1,719 (76.0)	4,323 (80.2)	< 0.001
**Change in HbA**_**1c**_ **between last pre-index and last post-index HbA**_**1c**_ **measurement**	Valid n	6,042	1,719	4,323	
	Mean (SD)	-0.8* (1.6)	-2.1* (2.0)	-0.3* (1.1)	< 0.001
Patients with HbA_1c_ decrease ≥ 1.0%, n (%)		2,326 (38.5)	1,259 (73.2)	1,067 (24.7)	< 0.001
Days from last pre-index to last post-index HbA_1c_ measurement, mean (SD)		321.4 (114.4)	309.5 (117.2)	326.1 (112.9)	< 0.001
**Patients with ≥ 90 days continuous treatment with OW sema T2D, n (%)**		5,628 (73.5)	1,568 (69.4)	4,060 (75.3)	< 0.001
**Among persistent patients, change in HbA**_**1c**_ **between the last pre-index and the last post-index HbA**_**1c**_ **measurements**	Valid n	3,748	993	2,755	
	Mean (SD)	-1.0* (1.5)	-2.5* (1.8)	-0.5* (1.0)	< 0.001
Patients with A1c decrease ≥ 1.0%, n (%)		1,585 (42.3)	812 (81.8)	733 (28.1)	< 0.001
Days from index date to last HbA_1c_ measurement, mean (SD)		251.4 (80.3)	245.1 (81.6)	253.7 (79.7)	0.004

* Ho: β = 0; p < 0.001

*p < 0.05

*p < 0.05, GLP-1 RAs, glucagon-like peptide-1 receptor antagonists; SGLT-2, sodium-glucose cotransporter-2 inhibitors; DPP-4s, dipeptidyl peptidase 4 inhibitors; TZDs, thiazolidinedione

## Discussion

Study patients had a mean decrease in HbA_1c_ of 0.8%; patients with uncontrolled diabetes saw a greater mean reduction in HbA_1c_ compared to those with controlled diabetes. Persistent patients with uncontrolled diabetes had a mean HbA_1c_ change of -2.5% (SD 1.8). While prescribing guidelines indicate that patients should initially be prescribed a 0.25/0.50 mg dose of OW sema T2D, 23.7% of patients in the study population were initially prescribed a 1.0 mg dose of OW sema T2D. However, it is possible that patients initially received their medication through sample pens that were not captured in claims data, which could result in misclassification of index dose. Additionally, many patients in this study (40.4%) were previously experienced with GLP-1 RAs before their initial prescription of OW sema T2D, which may impact prescribing behavior of OW sema T2D. 63.5% of patients in the uncontrolled group were prescribed a maintenance dose of 0.25/0.50 mg. Although some of these patients achieved glycemic targets, study results suggest there is an opportunity to improve prescribing practices to achieve greater glycemic control. Prescribing guidelines at the time of this study indicated that patients who had not achieved desired glycemic control could be prescribed a 1.0 mg weekly dose, 29] and updated guidelines indicate that patients can now also be prescribed a 2.0 mg weekly dose if needed. While this analysis does not reveal whether uncontrolled diabetes is related to patient characteristics or prescribing practices, there is likely an opportunity to improve implementation of best prescribing practices among all providers.

Patients in this study were medically complex filling an average of 13.6 (SD 6.4) different classes of medications and 15.5 (SD 7.5) different compounds. This means that these patients were at risk for experiencing adverse events and drug-drug interactions, complicating their care [38]. Patients in this study appeared to be in later stages of T2DM with 80.5% having diabetes with complications; patients also experienced a range of comorbid conditions including disorders of lipid metabolism (88.6%), and hypertension (85.7%), further complicating care decisions [39]. Despite these challenges, patients in this study experienced significant reductions in HbA_1c_.

Study results demonstrate that OW sema T2D is an effective real-world treatment for the management of T2DM and add to other work that demonstrated the effectiveness of OW sema T2D at lowering HbA_1c_ values [28]. As part of the SUSTAIN program, the efficacy of OW sema T2D was evaluated in six phase IIIa trials and in four phase IIIb trials [17–27]. Across the SUSTAIN trials, OW sema T2D 1.0 mg was shown to reduce HbA_1c_ by 1.5–1.8%.^28^ More recently, the SUSTAIN-FORTE trial demonstrated that a 2.0 mg dose of OW sema T2D can reduce HbA_1c_ values by 2.1%.^27^ The present study demonstrates that OW sema T2D reduces HbA_1c_ values even when used in a clinical practice setting outside of clinical trials. This finding aligns with other RWE studies [33, 40–48]. In a UK study, patients prescribed semaglutide showed a reduction in HbA_1c_ across all subgroups [41]. In the present study, patients who had uncontrolled T2DM before initiating therapy with OW sema T2D were more likely to be GLP-1 naïve and had the largest change in HbA_1c_. This also aligns with the UK study, which found that GLP-1 RA naïve patients had the largest reduction in HbA_1c_ [41]. Hansen et al. also conducted a real-word evaluation of patients prescribed OW sema T2D in an out-patient clinic and found results similar those reported in clinical trials [42]. Other real-world studies conducted in the US similarly found that OW sema T2DM significantly reduced HbA_1c_ in study patients and that persistent patients had greater reductions in HbA_1c_ compared to the overall study sample [33]. As in the present study, Visaria et al. found an overall reduction in HbA_1c_ of -0.9% and found patients with a higher pre-index HbA_1c_ had greater HbA_1c_ reductions [33]. The findings in the present study that show greater change in HbA_1c_ among persistent patients align with these US data, providing further evidence of OW sema T2Ds effectiveness at reducing HbA_1c_ in the real-world. This study has several limitations. It was conducted in a large US managed care population and may not be representative of all patients with T2DM. Data on medication use was taken from pharmacy claims and patients may not have taken their medication as directed. Medications acquired as physician samples or through coupons or discount programs are not captured in claims data and were not included in this study. Lab data were not available for every patient; patients that did not have a pre-index HbA_1c_ value recorded were excluded. Change in HbA_1c_ pre- and post-index was measured only among patients who had at least one pre-index and at least one post-index HbA_1c_ value. Patients initiating OW sema T2D may have done so due to worsening diabetes and elevated HbA_1c_; as a result, some of the reduction in HbA_1c_ observed could be regression to the mean. Data related to patients’ social determinants of health and other factors influencing health equity or access to care were not available via claims. Finally, this study ended in 2020 and does not incorporate more recent data.

## Conclusions

OW sema T2D is an effective T2DM treatment in the real-world (HbA_1c_ reduction of -0.8%), especially for patients with uncontrolled diabetes (HbA_1c_ reduction of -2.1%). These results illustrate the benefit of OW sema T2D in the real world. Study results indicate that there is an opportunity to improve prescribing behavior related to maintenance dose to better align with best practices and achieve greater glycemic control among patients. Future work should build on this data, using more recent data and incorporating important social determinates of health data. More research is needed to understand the relationship between prescribing patterns, provider specialty, dose escalation, and HbA_1c_ values.
